# Supplementary material for: Nationwide analysis of sex differences in waiting times for cataract surgery in Sweden between 2010 and 2022
Source: Commun Med (Lond). 2025 Mar 4;5:60. doi: 10.1038/s43856-025-00782-1 (PMC11880556; doi:10.1038/s43856-025-00782-1)
Supplement: Supplementary file 2 — Supplementary Information [file 43856_2025_782_MOESM2_ESM.pdf]

Supplementary Information to

Nationwide analysis of sex differences in waiting times for cataract surgery in Sweden between 2010 and 2022

Philip Jute, Gustav Stålhammar[1,2]

[1]St. Erik Eye Hospital, Stockholm, Sweden
[2]Department of Clinical Neuroscience, Division of Eye and Vision, Karolinska Institutet, Stockholm, Sweden

Table of contents

Supplementary Figure 1 2

Supplementary Method: Sensitivity Analyses 3

Supplementary Table 1 4

Supplementary Table 2 5

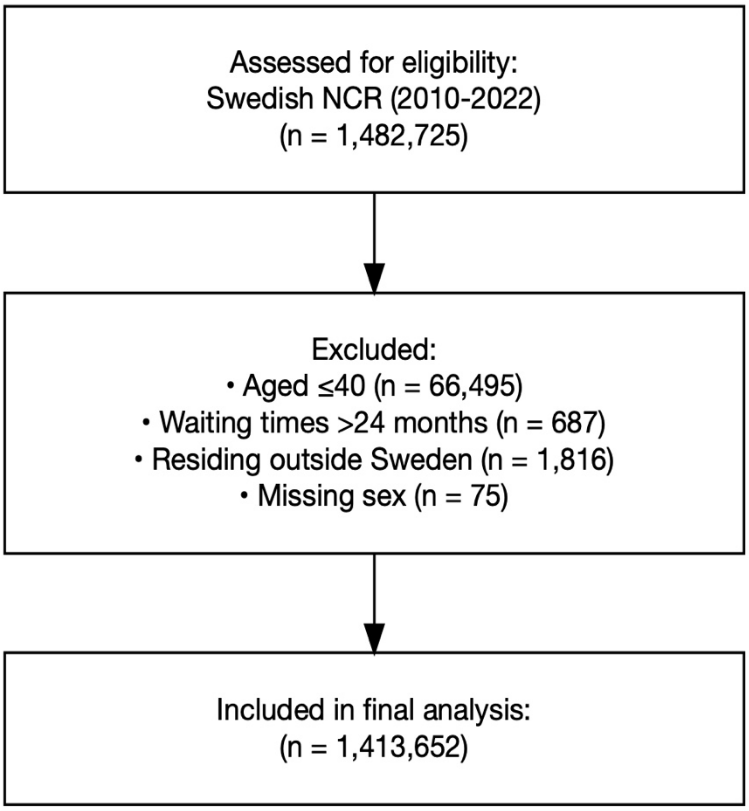

Supplementary Figure 1

Flowchart of the study population selection from the Swedish National Cataract Register (NCR) between 2010 and 2022. Patients were excluded if they were 40 years old or younger, had waiting times longer than 24 months, resided outside Sweden, or had missing sex information. The final cohort comprised those meeting all inclusion criteria.

Supplementary Method: Sensitivity Analyses

Two sensitivity analyses were conducted to evaluate potential factors contributing to waiting time outliers. The primary outcome measure was the comparison of waiting times between males and females in each subgroup, analyzed using the Mann-Whitney U test to assess statistical significance. Additionally, a Cox regression model was applied to identify predictors of waiting time among excluded patients.

1. Patients with waiting times exceeding 24 months:

This analysis included the 687 patients who were excluded for having exceptionally long waiting times (>24 months). The Mann-Whitney *U* test revealed a statistically significant difference in waiting times between males and females in this group ($P=8.73\times10^{-12}$), with females generally experiencing shorter waiting times compared to males.

Cox regression analysis was performed to explore the predictors of waiting time in this group. Among the predictors examined, significant associations were found for age ($P<2\times10^{-16}$), corneal guttata ($P=0.040$), diabetes ($P=0.003$), and macular disease ($P=0.006$). For instance, the presence of corneal guttata was associated with a 30% reduction in the hazard of shorter waiting times (HR 0.70, 95% CI 0.50–0.98, **Supplementary Table 1**). These findings suggest that patient-specific factors, rather than systematic sex-based differences, may explain the long waiting times in this subgroup.

2. Patients aged 40 years or younger:

This analysis included 66,495 patients who were excluded for being ≤40 years at the time of admission for cataract surgery, a subgroup typically associated with congenital, juvenile, or secondary cataract conditions. The Mann-Whitney *U* test demonstrated a significant difference in waiting times between males and females in this group ($P<2.2\times10^{-16}$), with females experiencing longer delays. For example, the mean waiting time for females was 74.1 days (SD 68.4) compared to 77.6 days (SD 87.2) for males.

Combined Cohort Analysis:

The impact of including the excluded patients on the primary findings was assessed using the combined cohort, which encompassed all patients, including those excluded from the primary analysis. The mean waiting time was 65.1 days (standard deviation [SD] 103) for females and 61.1 days (SD 98) for males, with a statistically significant difference between the sexes (*P*<2.2×10–16, Mann-Whitney *U* test). These results demonstrate that sex-based differences in waiting times persisted in the combined cohort, aligning with the findings from the primary analysis.

Conclusion:

The sensitivity analyses confirm that significant sex-based differences in waiting times persist in the younger patient subgroup, whereas the differences in the long-delay group appear to be explained by specific patient factors rather than sex. The inclusion of excluded patients does not alter the main conclusions, reinforcing the robustness of the primary findings.

Variable	B	S.E.	Wald Test	*P**	Exp(B)	95% Confidence Interval
Sex (Male)	0.09	0.03	3.62	<0.001	1.10	0.04–0.14
Age†	-0.04	<0.001	-48.55	<0.001	0.96	-0.04– -0.04
Pseudoexfoliations	-0.07	0.10	-0.71	0.48	0.93	-0.27–0.13
Cornea Guttata	-0.35	0.17	-2.05	0.04	0.70	-0.69– -0.02
Diabetes, type I or II	0.15	0.05	2.93	0.003	1.16	0.05–0.25
Macular Disease, any type	-0.19	0.07	-2.74	0.006	0.83	-0.32– -0.05
Glaucoma, any type	0.09	0.06	1.46	0.14	1.10	-0.03–0.22

*Bonferroni correction was applied to the *P* values to adjust for multiple comparisons. †Patient age at the time of admission for cataract surgery. S.E., standard error.

Supplementary Table 1. Multivariate Cox Regression: Predictors of Time to Cataract Surgery among excluded patients

01 - Stockholm lan
03 - Uppsala lan
04 - Sodermanland
05 - Ostergotland
06 - Jonkoping
07 - Kronoberg
08 - Kalmar
09 - Gotland
10 - Blekinge
12 - Skane
13 - Halland
14 - Vastra Gotaland
17 - Varmland
18 - Orebro
19 - Vastmanland
20 - Dalarna
21 - Gavleborg
22 - Vasternorrland
23 - Jamtland
24 - Vasterbotten
25 - Norrbotten

Supplementary Table 2. Regional Codes for Swedish Healthcare Areas
