# Supplementary material for: Nationwide analysis of sex differences in waiting times for cataract surgery in Sweden between 2010 and 2022
Source: Commun Med (Lond). 2025 Mar 4;5:60. doi: 10.1038/s43856-025-00782-1 (PMC11880556; doi:10.1038/s43856-025-00782-1)
Supplement: Supplementary file 3 — Description of Additional Supplementary Files [file 43856_2025_782_MOESM3_ESM.pdf]

Description of Additional Supplementary Files

File name: Supplementary Data

File description: Source data for all figures
