# Supplementary material for: Nationwide analysis of sex differences in waiting times for cataract surgery in Sweden between 2010 and 2022
Source: Commun Med (Lond). 2025 Mar 4;5:60. doi: 10.1038/s43856-025-00782-1 (PMC11880556; doi:10.1038/s43856-025-00782-1)
Supplement: Supplementary file 5 — REPORTING SUMMARY [file 43856_2025_782_MOESM5_ESM.pdf]

nature portfolio

Corresponding author(s): Gustav Stålhammar

Last updated by author(s): Feb 14, 2025

Reporting Summary

Nature Portfolio wishes to improve the reproducibility of the work that we publish. This form provides structure for consistency and transparency in reporting. For further information on Nature Portfolio policies, see our Editorial Policies and the Editorial Policy Checklist.

Statistics

For all statistical analyses, confirm that the following items are present in the figure legend, table legend, main text, or Methods section.

n/a	Confirmed	
☐	☒	The exact sample size (*n*) for each experimental group/condition, given as a discrete number and unit of measurement
☐	☒	A statement on whether measurements were taken from distinct samples or whether the same sample was measured repeatedly
☐	☒	The statistical test(s) used AND whether they are one- or two-sided<br>*Only common tests should be described solely by name; describe more complex techniques in the Methods section.*
☐	☒	A description of all covariates tested
☐	☒	A description of any assumptions or corrections, such as tests of normality and adjustment for multiple comparisons
☐	☒	A full description of the statistical parameters including central tendency (e.g. means) or other basic estimates (e.g. regression coefficient) AND variation (e.g. standard deviation) or associated estimates of uncertainty (e.g. confidence intervals)
☐	☒	For null hypothesis testing, the test statistic (e.g. *F*, *t*, *r*) with confidence intervals, effect sizes, degrees of freedom and *P* value noted<br>*Give P values as exact values whenever suitable.*
☒	☐	For Bayesian analysis, information on the choice of priors and Markov chain Monte Carlo settings
☒	☐	For hierarchical and complex designs, identification of the appropriate level for tests and full reporting of outcomes
☐	☒	Estimates of effect sizes (e.g. Cohen's *d*, Pearson's *r*), indicating how they were calculated

Our web collection on statistics for biologists contains articles on many of the points above.

Software and code

Policy information about availability of computer code

Data collection	Data for this study were retrieved from the Swedish National Cataract Register (NCR), established in 1992 to document all cataract surgeries performed nationwide. The register is governed by a steering committee comprising physicians representing both public and private healthcare sectors, academia, one nurse, and one patient representative. The NCR captures approximately 93% of all cataract surgeries conducted in Sweden, with data reliability continuously monitored and validated.<br>Patients eligible for inclusion were those over 40 years old undergoing a first-eye cataract operation between January 1, 2010, and December 31, 2022, following methods outlined in a previous study on cataract surgeries conducted in 2010 (n=1,482,725).<br>Patients aged 40 years or younger (n=66,495) were excluded, as cataracts in this group are typically congenital, juvenile, or secondary to other diseases or trauma, meaning standard waiting time rules do not apply. Additionally, patients with waiting times over 24 months (n=687) were excluded, as such extended delays are uncommon. These long waiting periods in the Swedish National Cataract Register (NCR) are likely due to registration errors or specific circumstances, such as a patient request for surgery by a particular surgeon.<br>Thirdly, 1816 patients residing outside Sweden were excluded, as clinicopathological data may be less reliable for these, and their waiting time for surgery may be influenced by factors non-typical to the standard situation in the Swedish healthcare system. Lastly, 75 patients without a recorded sex was excluded, leaving 1,413,652 patients for analysis.
Data analysis	All statistical analyses were conducted using IBM SPSS Statistics (version 29, Armonk, NY), GraphPad Prism (version 10.0.2, San Diego, CA, USA), and R (R Core Team, Vienna, Austria, 2022), with relevant packages including dplyr, ggplot2, tidyr, knitr, survminer, and survival.

For manuscripts utilizing custom algorithms or software that are central to the research but not yet described in published literature, software must be made available to editors and reviewers. We strongly encourage code deposition in a community repository (e.g. GitHub). See the Nature Portfolio guidelines for submitting code & software for further information.

Data

Policy information about availability of data

All manuscripts must include a data availability statement. This statement should provide the following information, where applicable:

- Accession codes, unique identifiers, or web links for publicly available datasets
- A description of any restrictions on data availability
- For clinical datasets or third party data, please ensure that the statement adheres to our policy

Patient-level data analyzed in this study are available from the Swedish National Cataract Register (https://rcsyd.se/anslutna-register/nationella-kataraktregistret). Access to these data requires approval from the Swedish Ethical Review Authority and the Swedish National Cataract Register's record keeper. Requests for data can be submitted through the register's website and must comply with Swedish regulations governing the use of healthcare data for research purposes.

Research involving human participants, their data, or biological material

Policy information about studies with human participants or human data. See also policy information about sex, gender (identity/presentation), and sexual orientation and race, ethnicity and racism.

Reporting on sex and gender	This study investigates sex-based differences in waiting times between admission for cataract surgery and the surgery itself. The primary objectives are to determine whether such differences exist, how they evolve over time, and whether they can be explained by other clinical variables. In this analysis, sex is used for categorization, as the data pertains to biological attributes rather than socially constructed groups. The distinction between sex and gender is important: sex refers to biological characteristics, while gender is shaped by social and cultural contexts.<br><br>Sex was considered during the study design phase and was determined through a combination of self-reporting and assignment via personal identity numbers in the Swedish public system, where only male and female sex designators are possible. Consent was not obtained for sharing individual-level data. The dataset comprised a total of 1.4 million individuals, categorized as female and male.
Reporting on race, ethnicity, or other socially relevant groupings	No data on race, ethnicity, or other social groupings were collected for this study. While it is possible that sex-based differences in waiting times could vary across ethnic or social groups, such analyses were not conducted due to the lack of available data in the source. Additionally, the primary objective was to investigate whether sex-based differences exist in general, rather than within specific populations.
Population characteristics	A total of 1,413,652 patients were included in the study, of whom 828,515 (59%) were female. Males had slightly better BCVA in the non-operated eye and were more likely to receive multifocal intraocular lenses (IOLs). Capsular tension rings were more commonly used, and postoperative endophthalmitis occurred more frequently in male patients, while pseudoexfoliations were more common among females.<br>The average waiting time from preoperative assessment to surgery was 64 days for females (standard deviation [SD] 126) and 60 days for males (SD 102). Differences in waiting times between females and males were observed across all visual acuity groups of the surgery eye, with females consistently experiencing longer average waiting times. These groups, categorized by visual acuity (≤0.1, 0.2, 0.3, 0.4, 0.5, 0.6, 0.7, 0.8, 0.9, and ≥1.0 on the decimal scale, which is equivalent to ≤20/200, 20/100, 20/66, 20/50, 20/40, 20/33, 20/28, 20/25, 20/22, and ≥20/20 on the Snellen scale, and 1.0, 0.7, 0.52, 0.40, 0.30, 0.22, 0.15, 0.10, 0.05, and 0.0 on the LogMAR scale.), demonstrated statistically significant disparities. For instance, in the ≤0.1 group, females had an average waiting time of 63 days (SD 71), compared to 57 days (SD 66 days) for males, a difference of 7 days. Similar differences were evident across all other groups. The magnitude of differences ranged from 2 days (SE 18, P<0.001) in the BCVA 0.7 group, to 7 days (SE 18, P<0.001) in the BCVA ≤0.1 group.
Recruitment	Participants for this study were drawn from the Swedish National Cataract Register (NCR), a comprehensive database established in 1992 to document all cataract surgeries performed across Sweden. The NCR captures approximately 93% of all cataract surgeries nationwide, encompassing both public and private healthcare providers. Data reliability is maintained through continuous monitoring and validation processes. The register is governed by a steering committee consisting of representatives from clinical practice, academia, nursing, and patient advocacy groups.<br><br>Eligible participants included individuals over 40 years old who underwent a first-eye cataract operation between January 1, 2010, and December 31, 2022. Exclusions were made for patients under 40 years of age (n=66,495) to avoid confounding by cataracts with distinct etiologies (e.g., congenital, juvenile, or secondary to trauma or systemic disease) where standard waiting time policies do not apply. Additionally, patients with waiting times exceeding 24 months (n=687) were excluded due to the likelihood of registration errors or exceptional circumstances, such as patient requests for specific surgeons.<br><br>Although the NCR provides comprehensive coverage, self-selection bias may exist, as patients who undergo surgery are likely more proactive in seeking care compared to those who do not. Additionally, disparities in healthcare access or regional differences in referral practices may influence results. However, the high capture rate of surgeries in the NCR minimizes the impact of these biases, ensuring that findings reflect a broad representation of cataract surgery patients in Sweden. The focus on patients undergoing surgery also means that the study is not designed to capture sex-based differences in unmet needs or access to care prior to surgery.
Ethics oversight	Swedish Ethical Review Authority

Note that full information on the approval of the study protocol must also be provided in the manuscript.

Field-specific reporting

Please select the one below that is the best fit for your research. If you are not sure, read the appropriate sections before making your selection.

☒ Life sciences ☐ Behavioural & social sciences ☐ Ecological, evolutionary & environmental sciences

For a reference copy of the document with all sections, see nature.com/documents/nr-reporting-summary-flat.pdf

Life sciences study design

All studies must disclose on these points even when the disclosure is negative.

Sample size

No formal sample size calculation was performed for this study. Instead, all eligible patients who met the inclusion criteria from the Swedish National Cataract Register (NCR) between January 1, 2010, and December 31, 2022, were included in the analysis. This approach ensured the largest possible sample size, encompassing 1,482,725 patients who underwent a first-eye cataract operation during the study period, after excluding individuals under 40 years of age and those with waiting times exceeding 24 months.

The decision to include the entire available dataset provides sufficient power to detect even small differences in waiting times between sexes and minimizes the risk of type II errors. The large sample size also enhances the generalizability of the findings, as it reflects the comprehensive, nationwide data captured by the NCR, covering approximately 93% of all cataract surgeries performed in Sweden. This robust dataset enables a detailed analysis of sex-based differences while accounting for potential variability across the population.

Data exclusions

To ensure the analysis focused on a relevant and comparable patient population, specific exclusions were applied to the dataset retrieved from the Swedish National Cataract Register (NCR). Patients under 40 years of age (n=66,495) were excluded, as cataracts in this age group are typically congenital, juvenile, or secondary to other diseases or trauma, and therefore not subject to standard waiting time practices. Additionally, patients with waiting times exceeding 24 months (n=687) were excluded, as such extended delays are rare and likely attributable to registration errors or exceptional circumstances, such as patient requests for surgery by a specific surgeon.

These exclusions were implemented to reduce potential biases and ensure that the analysis focused on a cohort representative of standard cataract surgery cases, thereby improving the validity of the findings regarding sex-based differences in waiting times. The final dataset included 1,482,725 patients who underwent a first-eye cataract operation between January 1, 2010, and December 31, 2022.

Replication

This study utilized real-world clinical data from the Swedish National Cataract Register (NCR), which captures approximately 93% of all cataract surgeries performed nationwide. Given the comprehensive and retrospective nature of this dataset, the findings cannot be directly replicated, as they rely on clinical data from a specific population and time frame (2010–2022). However, the results can be compared with previous studies to assess their consistency and plausibility.

The sex-based differences observed in this study align with findings from other research in similar healthcare settings, suggesting the results are realistic and representative. While replication of this exact dataset is not possible, future studies in other regions or countries with similar registers could evaluate whether comparable patterns are observed, further validating the findings.

Randomization

Allocation in this study was not randomized, as the primary focus was to analyze waiting times based on sex. Participants were categorized by sex, which was determined through self-reporting and personal identity numbers in the Swedish public system. Covariates were compared between the groups using descriptive statistics to identify any differences that could influence waiting times.

To account for potential confounding factors, multivariate regression analyses were conducted, adjusting for relevant clinical variables. This approach ensures that observed differences in waiting times are not solely attributable to variations in these covariates, allowing for a more accurate assessment of sex-based differences. While the lack of randomization may introduce inherent biases, the use of comprehensive statistical adjustments helps mitigate their potential impact on the findings.

Blinding

Blinding was not possible in this study, as allocation was based on sex, which was explicitly recorded in the raw data obtained from the Swedish National Cataract Register. Sex information was integral to the study’s primary objective of analyzing sex-based differences in waiting times and was therefore known to those handling and analyzing the data. While the lack of blinding could introduce potential observer bias, the use of standardized data collection procedures and comprehensive statistical methods minimizes the risk of such bias influencing the results.

Reporting for specific materials, systems and methods

We require information from authors about some types of materials, experimental systems and methods used in many studies. Here, indicate whether each material, system or method listed is relevant to your study. If you are not sure if a list item applies to your research, read the appropriate section before selecting a response.

Materials & experimental systems

n/a	Involved in the study
☒	☐ Antibodies
☒	☐ Eukaryotic cell lines
☒	☐ Palaeontology and archaeology
☒	☐ Animals and other organisms
☐	☒ Clinical data
☒	☐ Dual use research of concern
☒	☐ Plants

Methods

n/a	Involved in the study
☒	☐ ChIP-seq
☒	☐ Flow cytometry
☒	☐ MRI-based neuroimaging

Clinical data

Policy information about clinical studies
All manuscripts should comply with the ICMJE guidelines for publication of clinical research and a completed CONSORT checklist must be included with all submissions.

Clinical trial registration

Not a clinical trial. This is a retrospective register-based study.

Study protocol

This is not a clinical trial .and no protocol was registered in advance

Data collection

Participants for this study were drawn from the Swedish National Cataract Register (NCR), a comprehensive database established in 1992 to document all cataract surgeries performed across Sweden. The NCR captures approximately 93% of all cataract surgeries nationwide, encompassing both public and private healthcare providers. Data reliability is maintained through continuous monitoring and validation processes. The register is governed by a steering committee consisting of representatives from clinical practice, academia, nursing, and patient advocacy groups.

Eligible participants included individuals over 40 years old who underwent a first-eye cataract operation between January 1, 2010, and December 31, 2022. Exclusions were made for patients under 40 years of age (n=66,495) to avoid confounding by cataracts with distinct etiologies (e.g., congenital, juvenile, or secondary to trauma or systemic disease) where standard waiting time policies do not apply. Additionally, patients with waiting times exceeding 24 months (n=687) were excluded due to the likelihood of registration errors or exceptional circumstances, such as patient requests for specific surgeons.

Although the NCR provides comprehensive coverage, self-selection bias may exist, as patients who undergo surgery are likely more proactive in seeking care compared to those who do not. Additionally, disparities in healthcare access or regional differences in referral practices may influence results. However, the high capture rate of surgeries in the NCR minimizes the impact of these biases, ensuring that findings reflect a broad representation of cataract surgery patients in Sweden. The focus on patients undergoing surgery also means that the study is not designed to capture sex-based differences in unmet needs or access to care prior to surgery.

Outcomes

The primary outcome of this study was predefined as the waiting time between admission for cataract surgery and the surgery itself. This decision was informed by previous studies suggesting the potential for sex-based differences in waiting times. By focusing on this outcome, the study aimed to explore whether such differences exist, how they evolve over time, and whether they can be explained by other clinical variables. This predefined outcome aligns with the study's objectives and ensures comparability with earlier research in the field.

Plants

Seed stocks

No plants were studied

Novel plant genotypes

Describe the methods by which all novel plant genotypes were produced. This includes those generated by transgenic approaches, gene editing, chemical/radiation-based mutagenesis and hybridization. For transgenic lines, describe the transformation method, the number of independent lines analyzed and the generation upon which experiments were performed. For gene-edited lines, describe the editor used, the endogenous sequence targeted for editing, the targeting guide RNA sequence (if applicable) and how the editor was applied.

Authentication

Describe any authentication procedures for each seed stock used or novel genotype generated. Describe any experiments used to assess the effect of a mutation and, where applicable, how potential secondary effects (e.g. second site T-DNA insertions, mosiacism, off-target gene editing) were examined.
